# ICU requirements

**DOI:** 10.1007/s00068-025-02821-x

**Published:** 2025-03-16

**Authors:** Zsolt Balogh, Frank Hildebrand

**Affiliations:** 1 Department of Traumatology, John Hunter Hospital and University of Newcastle, Newcastle, NSW Australia; 2 Department of Orthopaedics, Trauma and Reconstructive Surgery, RWTH Aachen University, Aachen, Germany

**Keywords:** ESTES, Polytrauma, Whitebook

## Abstract

This section outlines the essential requirements for managing trauma patients in ICUs across Europe. It emphasizes the need for ICU accreditation at the highest national level and highlights criteria, including staffing, equipment, training programmes, protocols, and documentation for quality control. Key requirements encompass 24/7 admission capability, trained staff, multidisciplinary rounds, specialised observation beds, organ donation programmes, and participation in trauma resuscitations and hospital disaster planning. Desirable criteria, such as education, research activities, trauma protocol development, cross-rotation training, outreach services, and combined team training are also discussed, focused on fostering collaboration between trauma and intensive care services to ensure comprehensive trauma management.

## Introduction

The management of trauma patients in ICUs should adhere to the highest ICU designation category within each country, as regulated and standardised by the relevant professional bodies governing intensive or critical care. Although practices and regulation vary across Europe, the shared goal is to deliver the highest quality of care for trauma patients.

## Essential criteria

To ensure optimal care for trauma patients in the ICU, the following essential criteria must be met:

- **ICU accreditation**: Adherence to the highest national standards for ICU accreditation.
- **Staffing**: Adequate medical, nursing, and allied health personnel.
- **Essential equipment**: Availability of critical care tools and technologies.
- **Training Programmes**: Comprehensive ICU medical training for all staff.
- **Protocols and procedures**: Implementation of general ICU guidelines and policies.
- **Documentation**: Systems for quality control, such as trauma registries and audits.

Additional trauma-specific criteria include:

- **24/7 capability**: Continuous admission capability with a no-refusal policy for trauma cases.
- **Training**: All ICU staff must be ABCDE-trained to manage trauma patients effectively.
- **Multidisciplinary rounds**: Regular collaboration between ICU and trauma service staff (especially the trauma surgeons).
- **High-dependency observation beds**: Dedicated beds for high-risk, non-ventilated trauma patients, including those with solid organ injuries, spinal cord injuries, traumatic brain injuries, severe chest injuries, free flaps, and major postoperative cases.
- **Organ donation programmes**: Active participation in organ procurement, with established links to transplantation units.
- **Trauma resuscitations**: ICU doctors must participate in trauma resuscitations to provide timely interventions.
- **Trauma committee representation**: An ICU representative should liaise with the hospital trauma committee.
- **Isolation rooms**: Available for infectious diseases to prevent cross-contamination.
- **Advanced interventions**: Protocols and equipment for life-support interventions (e.g., extracorporeal membrane oxygenation (ECMO), renal replacement therapy).
- **Non-invasive ventilation**: Availability of equipment and protocols to support respiratory function.
- **Procedure protocols**: Standardised guidelines for common ICU procedures, such as tracheostomy, vascular line placement, chest tube insertion, and open abdomen management.
- **Nutrition protocols**: Implementation of enteral and parenteral nutrition.
- **Nosocomial infection control**: Prevention and surveillance protocols, along with antibiotic stewardship programmes, to minimise healthcare-associated infections.
- **Delirium management**: Screening protocols and prevention strategies to manage ICU delirium.
- **Point-of-care testing**: Real-time blood gas and coagulation monitoring capabilities.
- **Disaster planning**: Active involvement in hospital-wide disaster preparedness exercises and cross-hospital mass casualty responses.
- **Transport protocols**: Clear guidelines for intra- and inter-hospital trauma patient transfers.
- **Venous thrombosis prophylaxis**: Protocols to prevent thromboembolic complications.
- **Rapid-response system**: ICU participation in the hospital-wide rapid emergency care efforts, including the management of deteriorating non-ICU patients.

## Desirable criteria

While essential criteria ensure baseline care, the following desirable criteria can enhance ICU services:

- **Education activities**: ICU participation in trauma service training sessions to foster ongoing professional development.
- **Identifiable trauma lead**: A designated trauma lead from the ICU to streamline communication and coordination with trauma services.
- **Research**: Involvement in trauma service research activities to advance care practices and improve outcomes.
- **Protocol development**: Contributions to developing trauma-specific guidelines (e.g., massive transfusion and spinal clearance protocols).
- **Cross-rotation**: Training rotations between the ICU and trauma services to enhance interdisciplinary collaboration and balanced skills.
- **Outreach services**: Support for trauma wards managing high-risk discharged patients, helping to prevent complications and readmissions.
- **Team training**: Regular combined team training sessions for patient transport, trauma resuscitation, and management of deteriorating patients.
- **Faculty participation**: ICU staff serving as faculty in trauma training courses for broader healthcare education.

## Conclusion and needs for the future

The delivery of high-quality ICU care for trauma patients must balance international standards with local adaptations across Europe. Essential criteria, including staffing, equipment, and protocols, form the foundation for patient safety and optimal outcomes. Desirable criteria, such as research initiatives, cross-disciplinary collaboration, and advanced training programmes, further elevate the quality of care and foster innovation.

Geographical and systemic variations across European ICUs necessitate flexibility in implementation. For instance, anaesthesiologists may support trauma resuscitation teams in place of ICU physicians, or dual-role physicians may provide both anaesthesia and critical care. Such adaptations should be assessed individually to ensure they uphold patient safety and enhance trauma care delivery.

Looking ahead, collaboration among healthcare professionals, administrators, and policymakers is essential to harmonise practices and bridge disparities in trauma care. By building on shared knowledge and prioritising both foundational and aspirational standards, European ICUs can continue advancing the care of trauma patients, ensuring consistency and excellence across diverse healthcare systems.

## Data Availability

No datasets were generated or analysed during the current study.

